# Optimizing COVID-19 control with asymptomatic surveillance testing in a university environment

**DOI:** 10.1101/2020.11.12.20230870

**Published:** 2021-01-07

**Authors:** Cara E. Brook, Graham R. Northrup, Alexander J. Ehrenberg, IGI Testing Consortium, Jennifer A. Doudna, Mike Boots

**Affiliations:** 1 Department of Integrative Biology, University of California, Berkeley; 2 Center for Computational Biology, College of Engineering, University of California, Berkeley; 3 Innovative Genomics Institute, University of California, Berkeley; 4 Helen Wills Neuroscience Institute, University of California, Berkeley; 5 Memory and Aging Center, Weill Institute for Neurosciences, University of California, San Francisco; 6 Department of Molecular and Cell Biology, University of California, Berkeley; 7 College of Chemistry, University of California, Berkeley; 8 J. David Gladstone Institutes, San Francisco, CA; 9 Howard Hughes Medical Institute, University of California, Berkeley; 10 Department of Biosciences, University of Exeter, Penryn, UK

## Abstract

The high proportion of transmission events derived from asymptomatic or presymptomatic infections make SARS-CoV-2, the causative agent in COVID-19, difficult to control through the traditional non-pharmaceutical interventions (NPIs) of symptom-based isolation and contact tracing. As a consequence, many US universities have developed asymptomatic surveillance testing labs, to augment existing NPIs and control outbreaks on campus. We built a stochastic branching process model of COVID-19 dynamics to advise optimal control strategies in a university environment. Our model combines behavioral interventions in the form of group size limits to deter superspreading, symptom-based isolation, and contact tracing, with asymptomatic surveillance testing. We find that behavioral interventions offer a cost-effective means of epidemic control: group size limits of six or fewer greatly reduce superspreading, and rapid isolation of symptomatic infections can halt rising epidemics, depending on the frequency of asymptomatic transmission in the population. Surveillance testing can overcome uncertainty surrounding asymptomatic infections, with the most effective approaches prioritizing frequent testing with rapid turnaround time to isolation over test sensitivity. Importantly, contact tracing amplifies population-level impacts of all infection isolations, making even delayed interventions effective. Combination of behavior-based NPIs and asymptomatic surveillance also reduces variation in daily case counts to produce more predictable epidemics. Furthermore, targeted, intensive testing of a minority of high transmission risk individuals can effectively control the COVID-19 epidemic for the surrounding population. We offer this blueprint and easy-to-implement modeling tool to other academic or professional communities navigating optimal return-to-work strategies for the 2021 year.

## Introduction

Non-pharmaceutical interventions (NPIs) to control the spread of infectious diseases vary in efficacy depending on the natural history of pathogen that is targeted (1). Highly transmissible pathogens and pathogens for which the majority of onward transmission events take place prior to the onset of symptoms are notoriously difficult to control with standard public health approaches, such as isolation of symptomatic individuals and contact tracing (1). SARS-CoV-2, the causative agent in COVID-19, is a now a clear example of one of these difficult-to-control pathogens (2). While the first SARS-CoV was effectively contained via the isolation of symptomatic individuals following emergence in 2002 (3), at the time of writing, SARS-CoV-2 remains an ongoing public health menace that has infected more than 82 million people worldwide (4). Though the two coronaviruses are epidemiologically comparable in their basic reproduction numbers (R_0_) (3), SARS-CoV-2 has evaded control efforts largely because the majority of virus transmission events occur prior to the onset of clinical symptoms in infected persons (2)—in stark contrast to infections with the first SARS-CoV (3). Indeed, in many cases, SARS-CoV-2-infected individuals never experience symptoms at all (5–8) but, nonetheless, remain capable of transmitting the infection to others (9–13). Due to the challenges associated with asymptomatic and presymptomatic transmission (10), surveillance testing of asymptomatic individuals has the potential to play a critical role in COVID-19 epidemic control (14–16). Surveillance testing is always valuable for research purposes, but its efficacy as a public health intervention will depend on both the epidemiology of the focal infection and the characteristics of the testing regime. Here, we explore the effects of both behavior-based NPIs and asymptomatic surveillance testing on COVID-19 control in a university environment.

As the North American winter advances, the United States leads the globe with over 21 million reported cases of COVID-19 (4), and universities across the nation continue to struggle to control epidemics in their campus communities (17). To combat this challenge, colleges have adopted a variety of largely independent COVID-19 control tactics, ranging from entirely virtual formats to a mix of in-person and remote learning, paired with strict behavioral regulations, and—in some cases—in-house asymptomatic surveillance testing (18). As we approach the 2021 academic semester, asymptomatic surveillance testing is likely to play a key role in university plans for expanding reopening in the new semester (18, 19). In March 2020, shortly after the World Health Organization declared COVID-19 to be a global pandemic (20), the University of California, Berkeley, launched its own pop-up SARS-CoV-2 testing lab in the Innovative Genomics Institute (IGI) (21) with the aim of providing COVID diagnostic services to the UC Berkeley community and underserved populations in the surrounding East Bay region. Though the IGI RT-qPCR-based pipeline was initially developed to service clinical, symptomatic nasopharyngeal and oropharyngeal swab samples (21), the IGI subsequently inaugurated an asymptomatic surveillance testing program for the UC Berkeley community, through which—at the time of this writing—over 18,000 faculty, students, and staff in the UC Berkeley community have since been serviced with over 105,000 tests and counting (22).

Here we developed a stochastic, agent-based branching process model of COVID-19 spread in a university environment to advise UC Berkeley on best-practice approaches for surveillance testing in our community and to offer guidelines for optimal control in university settings more broadly. Previous modeling efforts have used similar approaches to advocate for more frequent testing with more rapid turnaround times at the expense of heightened test sensitivity (14, 15) or to weigh the cost-effectiveness of various testing regimes against symptom-based screening in closed university or professional environments (16). Our model is unique in combining both behavioral interventions with optimal testing design in a real-world setting, offering important insights into efficient mechanisms of epidemic control and an effective tool to optimize control strategies.

## Model design.

**Figure 1.**
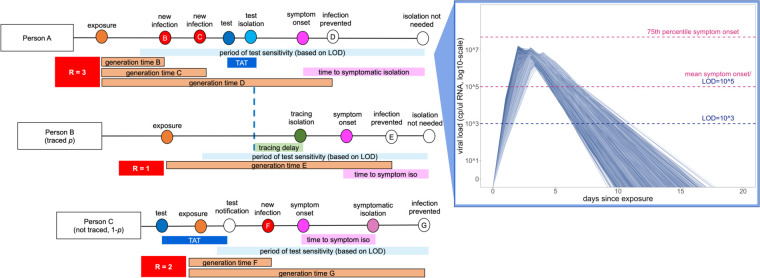
Conceptual schematic of branching process model of SARS-CoV-2 dynamics. Person A is isolated through testing after exposing Person B and Person C. Person B is then isolated through contact tracing, while Person C is not traced but is nonetheless ultimately isolated through symptomatic surveillance. A viral titer trajectory (right) is derived from a within-host viral kinetics model (Supplementary File 1), yielding the mean titer trajectory and 95% confidence interval shown here. The 25^th^ and 75^th^ titer threshold percentile for the onset of symptoms are depicted in pink, such that 32% of individuals modeled in our simulations did not present symptoms. Schematic is adapted in concept from Hellewell et al. (2020) (25).

Our model takes the form of a stochastic branching process model, in which a subset population of exposed individuals (0.5%, derived from the mean percentage of positive tests in our UC Berkeley community (22)) is introduced into a hypothetical 20,000 person community that approximates the campus utilization goals for our university in spring 2021. With each timestep, the disease parameters for each infected case are drawn stochastically from distributions representing the natural history of the SARS-CoV-2 virus, paired with realistic estimates of the timeline of corresponding public health interventions (2, 16, 23) (Figure 1). Our flexible model (published here with open-access R-code (24)) allows for the introduction of NPIs for COVID-19 control in four different forms: (1) group size limits, (2) symptom-based isolations, (3) surveillance testing isolations, and (4) contact tracing isolations that follow after cases are identified through screening from symptomatic or surveillance testing. Because we focus our efforts on optimal surveillance testing regimes, we do not explicitly model other NPIs, such as social distancing and mask wearing; however, the effects of these behaviors are captured in our representation of R-effective (hereafter, R_E_) for both within-campus and out-of-campus transmission. We do not explicitly incorporate vaccination in the current analysis, but our open-access R-code is programmed to facilitate easy extension of our work to include exploration of the varied effects of NPIs on populations with a subset of vaccinated individuals (24), as these scenarios become more pervasive in the 2021 year.

R_E_ is the product of the pathogen basic reproduction number (R_0_) and the proportion of the population that is susceptible to disease. R_E_ is thus a dynamic value which corresponds to the number of new infections caused by a single infection at a given timepoint within a specified community. We compute an independent R_E_ for each infectious person in our population that is the combined result of both heterogeneity in individual infectiousness and heterogeneity in individual contact events that could result in transmission. To determine R_E_, we first draw a value of potential cases for each infectious individual in our population from the SARS-CoV-2 negative binomial distribution for R_0_, estimated to have a mean value of 2.5 and a dispersion parameter (*k*) of 0.10 (26). Though representation of R_E_ in log-normal vs. negative binomial form will not change the average number of cases generated per epidemic, the negative binomial distribution replicates the dynamics of superspreading events, which are known to play an important role in SARS-CoV-2 dynamics (27–32). Indeed, there is growing direct empirical evidence that COVID-19 epidemiology exhibits a negative binomial R_E_ across multiple systems (31, 33–35); as few as 10% of infectious individuals may be responsible for 80% of onward SARS-CoV-2 transmissions (36).

We next assume that a minority (10%) of possible onward transmissions are lost to the external community (e.g. an infectious UC Berkeley community member infects someone outside the UC Berkeley community), and we remove these from our model, such that we ultimately aim to report within-campus R_E_ as the number of onward cases that a single infectious university community member causes within the university community (Supplementary File 1). To achieve this, we next assume that social distancing, masking, and behavioral modifications in our community will modulate dynamics such that some of the remaining 90% of the original R_0_-derived potential infections do not take place. Because we are specifically interested in advising UC Berkeley on group size limits for gatherings, we next draw a number of possible onward transmission events for each infectious individual from a simple Poisson distribution with *λ* = 3, signifying the average number of possible encounters (i.e. cross-household dining, shared car rides, indoor meetings, etc.) per person that could result in transmission. We then use published estimates of the generation time of onward transmission events for SARS-CoV-2 infection (2) to draw event times for these encounters, and we distribute each infectious person’s original number of R_0_-derived potential cases among these events at random. This ensures that multiple transmissions are possible at a single event; the most extreme superspreading events occur when persons with heterogeneously high infectiousness draw a large number of potential cases, which are concentrated within a relatively small number of discrete transmission events. When group size limit NPIs are imposed, case numbers for each event are truncated at the intervention limit.

For each infectious individual, we additionally generate an independent virus trajectory, using a within-host viral kinetics model for SARS-CoV-2 upper respiratory tract infections, which is structured after the classic target cell model (37–40). From each independent virus trajectory, we can then infer a time-varying transmissibility, which is modeled as a Michaelis-Menten-like function of viral load (40). Deviating from the original published model, we fix the within-host viral kinetics model constant, *θ*, at a value that allows for a ~50% probability of infection occurring per transmissible contact event at an infectious individual’s peak viral load (40). Because all possible onward transmissions have been assigned an event generation time, we next evaluate the viral load of the infectious person at the time of each potential transmission to determine whether or not it actually takes place. By these metrics, our original R_0_-derived possible cases are halved, such that R_E_, the number of average onward infections caused by a single infectious person in the UC Berkeley community, is reduced to just over one (R_E_=1.05), consistent with published estimates of Bay Area R_E_ and initial asymptomatic test results in our community (22, 41). The majority of transmission events occur when the infectious host has higher viral titers, thus biasing new case generations towards earlier timesteps in an individual’s infection trajectory, as is realistic for COVID-19 (23) (Figure 1).

In addition to modulating the probability of onward transmission events, each infectious individual’s virus trajectory additionally allows us to compute a timing of symptom onset, which corresponds to the timepoint at which an individual’s virus trajectory crosses some threshold value for presentation of symptoms. We draw each threshold randomly from a log-normal distribution with a mean of 10^5^ virus copies per μl of RNA; by these metrics, roughly 32% of our modeled population presents as asymptomatic, in keeping with published estimates for SARS-CoV-2 (6, 7). Using each infectious individual’s viral load trajectory, we are next able to compute a period of test sensitivity, corresponding to the time during which viral load is high enough for detection by the virus test in question, based on the modeled limit of detection (LOD). Surveillance testing results in higher “false-negative” test results both very early and very late in infection when viral loads are below the LOD for the adopted assay (42) (Figure 1), though most tests should reliably detect infectious cases with viral titers >10^6^ cp/μl (43–45). We explore dynamics across a range of published values for test LOD: 10^1^, 10^3^, and 10^5^ virus copies per μl of RNA. The IGI’s RT-qPCR-based testing pipeline has a published sensitivity of 1 cp/μl (21), while the majority of SARS-CoV-2 RT-qPCR tests nationally are reliable above a 10^3^ cp/μl threshold (46); less-sensitive antigen-based and LAMP assays report detection limits around 10^5^ cp/μl (47, 48).

In addition to within-community transmissions, all individuals in the modeled population are also subjected to a daily hazard (0.15%) of becoming infected from an external source, based on published estimates of R_E_ and COVID-19 prevalence in Alameda County (41, 49). We report the mean results of 100 stochastic runs of each proposed intervention.

## Results.

### Comparing behavioral NPIs for COVID-19 control.

We first ran a series of epidemic simulations using a completely mixed population of 20,000 individuals subject to the infection dynamics outlined above to compare and contrast the impacts of our four NPIs on COVID-19 control. We introduced an initial population of 100 infectious individuals (0.5%) at timestep 0 and compared the effects of a single target intervention on epidemic trajectories after the first 50 days of simulation. Less intensive or intervention-absent scenarios allowed infectious cases to grow at unimpeded exponential rates, rapidly exhausting our susceptible supply and making it necessary to compare results at a consistent (and early) timepoint in our simulated epidemics.

**Figure 2.**
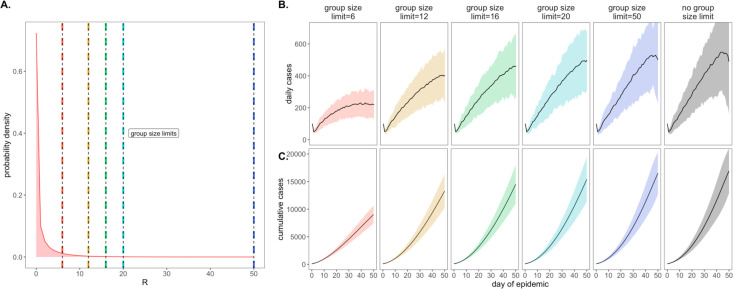
Effects of group size limits on COVID-19 dynamics. **A.** Negative binomial R_E_ distribution with mean = 1.05 and dispersion parameter (k) = 0.10. The colored vertical dashes indicate group size limits that ‘chop the tail’ on the R_E_ distribution; for 90% of the population, coincident cases allocated to the same transmission event were truncated at the corresponding threshold for each intervention. **B.** Daily new cases and, **C.** Cumulative cases, across a 50-day time series under corresponding, color-coded group size limits.

As a consequence of our representation of R_E_ in negative binomial form, we first considered the COVID-19 control effectiveness of group size limits on in-person gatherings, which doubled as upper thresholds in transmission capacity (Figure 2). Assuming that 90% of the modeled population adhered to assumed group size regulations, we found that limiting outdoor gatherings to groups of six or fewer individuals saved a mean of ~7,900 cases per 50-day simulation (in a 20,000 person population) and corresponded to an R_E_ reduction of nearly 0.20 (reducing R_E_ from 1.05 to subclinical 0.86; Figure 2; Supplementary File 2). By contrast, a large group size limit of 50 persons had almost no effect on epidemic dynamics; under published estimates of SARS-CoV-2 negative binomial R_E_ (26), a group size limit of 50 will restrict transmission from only 0.00039% of infectious individuals (Figure 2). Gains in epidemic control from group size limits resulted from avoidance of superspreading events, an approach that was effective for negative binomial but not log-normal representations of R_E_ that lack the transmission “tail” characteristic of a superspreader distribution (32) (Figure 2-S1). Importantly, by avoiding superspreading events, group size limits also reduced variance in daily case counts, yielding more predictable epidemics, which are easier to control through testing and contact tracing (2, 23, 25). Over the July 4 weekend, surveillance testing resources in our UC Berkeley community were overwhelmed and containment efforts challenged after a single superspreading event on campus (50).

**Figure 3.**
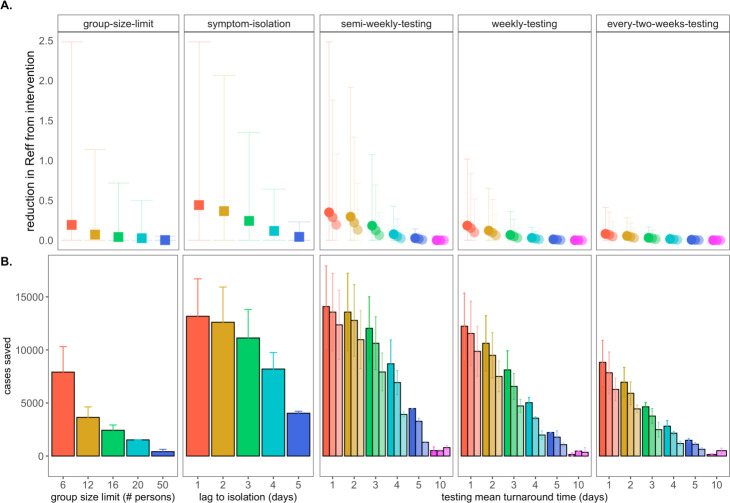
Impacts of NPIs on COVID-19 control. **A.** Mean reduction in R_E_* and **B.** cumulative cases saved across 50-day simulated epidemics under assumptions of differing non-pharmacological interventions (NPIs). NPIs are color-coded by threshold number of persons for group-size limits, lag-time for symptom-based isolations, and mean turnaround time from test positivity to isolation of infectious individuals for testing isolations. For testing isolations, shading hue corresponds to test limit of detection (LOD) with the darkest colors indicating the most sensitive tests with an LOD of 10^1^ virus copies/μl of RNA. Progressively lighter shading corresponds to LOD = 10^3^, 10^5^, and 10^7^ cp/μl. *Note: R_E_ reduction (panel A) is calculated as the difference in mean R_E_ in the absence vs. presence of a given NPI. The upper confidence limit (uci) in R_E_ reduction is calculated as the difference in uci R_E_ in the absence vs. presence of NPI. In our model, mean R_E_ in the absence of NPI equals 1.05 and uci R_E_ in the absence of NPI equals 8.6.

We next investigated the impacts of variation in lag time to self-isolation post-symptom onset for the just under 70% of individuals likely to present with COVID-19 symptoms in our modeled population (Figure 3). At UC Berkeley, all essential students, faculty, and staff must complete a digital ‘Daily Symptom Screener’ before being cleared to work on campus; here, we effectively model the delay post-initial symptom onset to the time at which each individual recognizes symptoms sufficiently to report to the Screener and isolate. For each infected individual in our population, we draw a symptom-based isolation lag from a log-normal distribution centered on a mean of one to five days, assuming the entire population to be compliant with the selected lag.

By these metrics, a rapid, one day lag in symptom-based isolation is the fourth-most effective intervention in our study, with a mean of more than 13,100 cases saved in a 50-day simulation (again, in a 20,000 person population), corresponding to an R_E_ reduction of 0.67, from 1 to 0.38 (Supplementary File 2). Longer lag times to isolation produced less dramatic results, but even an average five-day lag to isolation post-symptom onset nonetheless yielded more than 4,000 cases saved and reduced R_E_ by a mean of 0.06. The efficacy of symptom-based isolation decreased at higher virus titer thresholds for symptom onset, corresponding to a higher asymptomatic proportion (~50%) of the population (Figure 3-S1); some empirical findings suggest that these higher titer thresholds for symptom onset may more accurately reflect COVID-19 epidemiology (51). Because both group size limits and daily screening surveys to facilitate symptom-based isolation can be implemented without expending substantial resources, we advocate for these two approaches as particularly cost-effective COVID-19 control strategies for all university and small community environments—especially those lacking an on-site surveillance testing lab.

### Comparing surveillance testing NPIs for COVID-19 control.

Our primary motivation in developing this model was to advise UC Berkeley on best-practices for asymptomatic surveillance testing. As such, we focused efforts on determining the most effective use of testing resources by comparing surveillance testing across a range of approaches that varied test frequency, test turnaround time (TAT, the time from which the test was administered to the timing of positive case isolation), and test sensitivity (based on the LOD).

We compared all permutations of surveillance testing NPIs, varying test frequency across semi-weekly, weekly, and every-two-week regimes, investigating TAT across delays of one to five and ten days, and exploring LODs of 10^1^, 10^3^, and 10^5^ virus copies per μl of RNA. These test frequency regimes reflect those under consideration at UC Berkeley today: from August-December 2020, UC Berkeley undergraduates residing in university residence halls were subject to compulsory semi-weekly asymptomatic surveillance testing, while all other campus community members were permitted to take part in voluntary testing with a recommended weekly or every-two-week frequency. TAT values in our model reflect the reality in range of testing turnaround times from in-house university labs like that at UC Berkeley to institutions forced to outsource testing to commercial suppliers (52), and LOD values span the range in sensitivity of available SARS-CoV-2 tests (21, 46–48).

Across testing regimes broadly, we found test frequency, followed by TAT, to be the most effective NPIs, with LOD exerting substantially less influence on epidemic dynamics, consistent with findings published elsewhere (14, 15). The top three most effective NPIs in our study corresponded to semi-weekly testing regimes with one- and two-day TATs across 10^1^ and 10^3^ cp/μl LODs. These three scenarios yielded mean cases saved ranging from just over 14,000 to just over 13,500 in the first 50 days of simulation and produced an R_E_ reduction capacity between 0.97 and 0.80 (Figure 3; Supplementary File 2). Halving test frequency to a weekly regimen, under assumptions of TAT=1 and LOD=10^1^, resulted in a nearly 48% decrease in the NPI’s R_E_ reduction capacity. By comparison, a single extra day lag from one to two-day TAT under semi-weekly testing conditions at LOD=10^1^ cp/μl yielded a modest 16% decrease in R_E_ reduction capacity. However, longer delays in TAT of up to ten days or more—not unusual in the early stages of the COVID-19 pandemic (52)—were not significantly different from scenarios in which no intervention was applied at all. This outcome results from the rapid generation time of SARS-CoV-2 (2); most infectious individuals will have already completed the majority of subsequent transmissions by the time a testing isolation with a 10-day TAT is implemented. Nonetheless, encouragingly, reducing test sensitivity from 10^1^ to 10^3^ under a semi-weekly, TAT=1 regime decreased R_E_ reduction capacity by only 18%, offering support to advocates for more frequent but less sensitive tests (53) but also highlighting the added benefit incurred when university testing labs, like that at UC Berkeley, are able to provide both frequent and sensitive PCR-based testing.

Addition of a contact tracing intervention, in which 90% of infectious contacts were traced and isolated within a day of the source host isolation, to NPI scenarios already featuring either symptom-based or surveillance testing isolation enhanced each intervention’s capacity for epidemic control (Figure 3-S2). Of note, contact tracing boosted performance of some of the poorest performing testing interventions, such that even those previously ineffective surveillance regimens with 10-day TAT nonetheless averted cases and significantly reduced R_E_ when infectious contacts could be isolated. For a semi-weekly testing regime at LOD=10^1^ cp/μl and TAT=10 days, the addition of contact tracing increased mean cases saved from ~510 to >8,600 and increased R_E_ reduction capacity from 0.000080 to 0.27 (Supplementary File 3).

### Optimizing combined NPIs for COVID-19 control.

**Figure 4.**
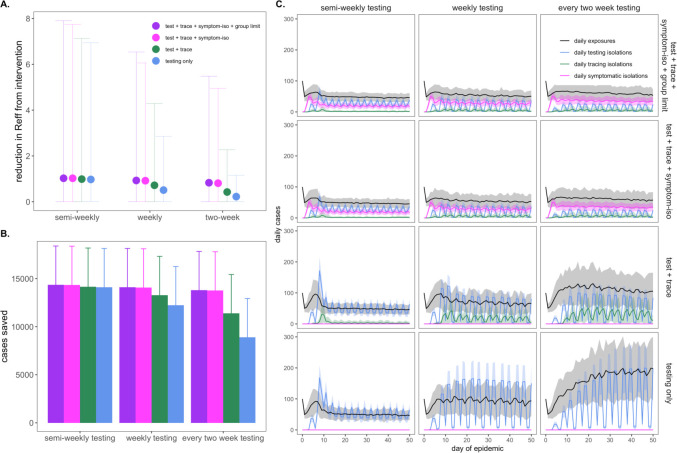
Combining behavioral and surveillance testing NPIs for COVID-19 control. **A.** Mean reduction in R_E_, **B.** cumulative cases saved, and **C.** daily case counts for the first 50 days of the epidemic, across regimes of differing testing frequency and a combination of surveillance testing, contact tracing, symptomatic isolation, and group size limit interventions. All scenarios depicted here assumed test TAT, symptomatic isolation lags, and contact tracing lags drawn from a log-normal distribution with mean=1. LOD was fixed at 10^1^ and group size limits at 12. Dynamics shown here are from biweekly testing simulations in which testing was limited to two test days per week.

Our modeled simulations indicate that it is possible to achieve largely equivalent gains in COVID-19 control from NPIs in the form of group size limits, symptom-based isolations, and surveillance testing isolations—though gains from symptom-based behavioral isolations are jeopardized under assumptions of a higher proportion of asymptomatic individuals (Figure 3-S1). Nonetheless, the most effective interventions are realized when behavioral control mechanisms are *combined* with surveillance testing (Figure 4). Assuming a one day TAT and 10^1^ cp/μl LOD, we found that adding (a) contact tracing with 90% adherence and a one-day lag, plus (b) symptom-based isolation with a one-day lag, plus (c) a group size limit of twelve persons to an every-two-week surveillance testing regimen could elevate the R_E_ reduction capacity from 0.22 to 0.83 and almost double the ~6,600 cases saved from the testing intervention alone (Supplementary File 4). Combining interventions enables less rigorous testing regimes to rival the effectiveness of semi-weekly surveillance testing without expending additional resources. In addition, combining interventions results in less variation in the cumulative case count, as many layers of opportunity for infection isolation help limit the likelihood of a superspreading event spiraling out of control (Figure 4-S1).

Following on this theme, we also experimented with varying the distribution of days allocated to surveillance testing, without changing the frequency with which each individual was tested. Specifically, we explored semi-weekly, weekly, and every-two-week testing regimens in which tests were administered across two, five, and seven available testing days per week. More broadly distributed test days corresponded to fewer tests per day at a population level but, as with more intervention layers, resulted in less variation in the cumulative total cases because testing isolations more closely tracked daily exposures (Figure 4-S1).

### Modeling COVID-19 dynamics in the campus community.

**Figure 5.**
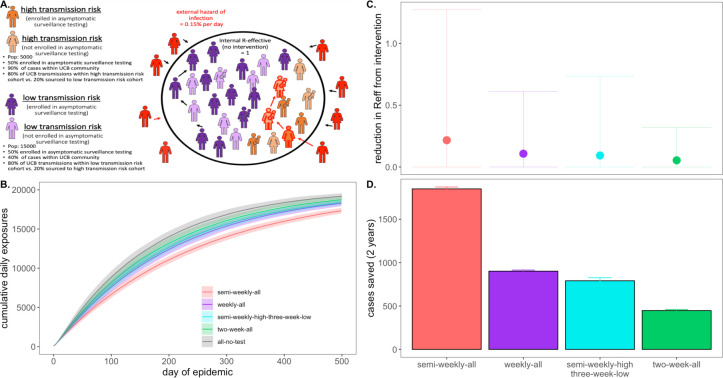
Targeted testing of high transmission risk cohorts in a heterogenous population. **A.** Schematic of transmission risk group cohorts in the heterogenous model. The population is divided into 5,000 “high transmission risk” and 15,000 “low transmission risk” individuals, for which, 90% and 40% of the proportion of transmission events take place within the UC Berkeley community, respectively. Of those transmission events within the Berkeley community, the majority (80%) are restricted within the same transmission risk group as the infector, while 20% are sourced to the opposing risk group. Half of each cohort is assumed to be enrolled in asymptomatic surveillance testing and subjected to the differing test frequency regimes depicted in panels **B.** through **D.** Panel **B**. shows the progression of cumulative cases across 730 days of simulation for each testing regime, while panel **C**. and **D**. give, respectively, the reduction in R_E_ and the total cases saved achieved by each test regime vs. a no intervention baseline.

In our final analysis, we sought to advise the IGI on surveillance testing strategies explicitly by simulating epidemics in a more realistic, heterogeneous population modeled after the UC Berkeley campus community (Figure 5). To this end, we subdivided our 20,000 person university population into a 5,000 person “high transmission risk” cohort and a 15,000 person “low transmission risk” cohort, assuming “high transmission risk” status to correspond to individuals (such as undergraduates), living in high density housing with a majority of contacts (90%) concentrated within the UCB community and “low transmission risk status” to correspond to individuals (such as faculty members or postdoctoral scholars) with only limited contacts (40%) in the UCB community. We imposed a 12-person group size limit (with 90% adherence) on the population as a whole, as recommended by the City of Berkeley Public Health Department in the early months of the pandemic (54), and assumed a one-day average lag in symptom-based isolation for all cohorts. To add additional realism, we enrolled only 50% of each transmission risk group in our modeled surveillance testing program (to mimic adherence—though surveillance testing is compulsory for undergraduates residing in residence halls at UC Berkeley (22)). We assumed that 95% efficacy in contact tracing (with a mean tracing delay of one day) for those enrolled in our surveillance program but only 50% efficacy for those not enrolled; UC Berkeley has encouraged all community members to enroll in the ‘CA Notify’ digital contract tracing app developed by Apple and Google (55). For all testing interventions, we assumed LOD=10^1^ cp/μl and TAT=2 days, the average for the IGI surveillance testing lab (21).

We found that targeted, semi-weekly testing of 50% of individuals in the high transmission risk cohort, paired with every-three-week testing of enrolled individuals in the low transmission risk cohort yielded mean R_E_ reduction and cumulative cases saved on par with that achieved from weekly testing (and better than that achieved from every-two-week testing) of all enrolled individuals in the population at large (Figure 5). Targeting the highest transmission-risk populations with testing surveillance allows practitioners to save valuable testing resources while simultaneously controlling the epidemic for the entire community. Importantly, while mean R_E_ reduction and cumulative cases were largely comparable between the targeted, semi-weekly testing regiment and the untargeted, weekly regimen, the observed variance in intervention efficacy (Figure 5C) was substantially greater for the targeted scenario, in which the low transmission risk cohort was only tested once every three weeks. This results from a higher probability that a rare superspreading event could occur in the infrequently monitored low transmission risk cohort, thus reaffirming our previous observation that more frequent surveillance testing regimens result in more predictable—and easier to control—epidemics.

Notably, irrespective of intervention, the diminished transmissibility of the “low transmission risk” population in this heterogeneous model structure greatly reduced epidemic spread in subsequent simulations as compared with those presented previously in the perfectly mixed environment; as a result, we here compared interventions after 500 days of simulation, rather than 50. The heightened realism of our heterogenous population generated slow-moving epidemics more closely resembling those we are currently witnessing in our university environment.

## Discussion.

We built a stochastic branching process model of SARS-CoV-2 spread in a university environment to advise UC Berkeley on best-practice strategies for effective asymptomatic surveillance in our pop-up IGI testing lab—and to offer a model for other institutions attempting to control the COVID-19 epidemic in their communities. While previous work has explored the isolated effects of specific NPIs—including group association limits (32), symptomatic isolation (2, 14–16, 23, 25), asymptomatic surveillance testing (14–16), and contact tracing (2, 23, 25)—on COVID-19 control, ours is the only model to date which investigates these interventions simultaneously and does so in a realistic and easily applicable setting. We offer an easy-to-implement modeling tool that can be applied in other educational and workplace settings to provide NPI recommendations tailored to the COVID-19 epidemiology of a specific environment.

Results from our analysis of behavior-based NPIs support previous work (2, 14–16, 23, 25, 32) in showing that stringent group size limitations to minimize superspreading events and rapid symptom-based isolations offer an effective means of epidemic control in the absence of surveillance testing resources. However, because of the unique natural history of the SARS-CoV-2 virus, for which the majority of transmission events result from asymptomatic or presymptomatic infections (2, 25), symptom-based NPIs cannot reduce epidemic spread completely, and small community environments will always remain vulnerable to asymptomatic case importation. Moreover, symptom-based NPIs pose less effective means of epidemic control under scenarios assuming a higher proportion of asymptomatic individuals; empirical evidence suggests that SARS-CoV-2 infection may result in asymptomatic infection in up to nearly 70% of the population in select environments (51). For this reason, our results emphasize the importance of asymptomatic surveillance testing to prevent ongoing epidemics in universities and other small community environments. As more data becomes available on both the proportion of asymptomatic infections and their contributions to SARS-CoV-2 transmission, the relative importance of group size interventions, symptom-based isolation, and asymptomatic surveillance testing in different epidemiological contexts will be possible to determine from our modeling framework.

As with behavioral interventions, our exploration of optimal surveillance testing regimes supports findings that have been published previously but with a few key extensions and critical novel insights. As has been recently highlighted (14, 15), we find that the most cases are saved under asymptomatic testing regimes that prioritize heightened test frequency and rapid turnaround time over test sensitivity. Importantly, we extend previous work to highlight how more rigorous testing regimes—and those combined with one or more behavioral interventions—greatly reduce variance in daily case counts, leading to more predictable epidemics. We find that the reduction in daily case variation is even more pronounced when test regimes of equivalent frequency are distributed more broadly in time (i.e. tests are offered across more days of the week), thus minimizing the likelihood of compounding transmission chains that may follow upon a superspreading event. Additionally, we demonstrate how a focused stringent testing regime for a subset of “high transmission risk” individuals can effectively control a COVID-19 epidemic for the broader community. Taken together, our model shows the utility of a multi-faceted approach to COVID-19 control and offers a flexible tool to aid in prioritization of interventions in different university or workplace settings.

Finally, our paper presents the only COVID-19 surveillance model published to date that combines asymptomatic testing with contact tracing, thus highlighting the compounding gains effected by these two interventions: contact tracing amplifies the control impacts of both symptom-based and surveillance testing-based isolations, such that even intervention scenarios assuming long delays in isolation after symptom onset or slow turnaround-times for test results can nonetheless greatly reduce the transmission capacity of COVID-19. These findings further emphasize the critical role that asymptomatic surveillance testing is likely to play in ongoing efforts to control COVID-19 epidemics into the 2021 year. Even limited surveillance testing may offer substantial gains in case reduction for university and workplace settings that already have efficient symptomatic isolation and contact tracing programs in place. Our model allows us to prioritize when and where these gains are most likely to be achieved.

Because we do not explicitly model SARS-CoV-2 transmission in a mechanistic, compartmental framework (56, 57), our analysis may overlook some more subtle insights into long-term disease dynamics. More complex analyses of interacting epidemics across larger spatial scales or investigations of vaccination delivery and the duration of immunity will necessitate implementation of a complete compartmental transmission model. However, our use of a stochastic branching process framework makes our model simple to implement and easily transferrable to other semi-contained small community environments, including a wide range of academic settings and workplaces (24). We make this tool available to others interested in exploring the impacts of targeted public health interventions—in particular, surveillance testing regimes—on COVID-19 control in more specific settings in the upcoming 2021 year. We at the University of California, Berkeley are committed to maintaining the safest campus environment possible for our community, using all intervention tools at our disposal. We advise those in similar positions at other institutions to employ the behavioral interventions outlined here, in concert with effective surveillance testing regimes, to reduce community epidemics of COVID-19 in the upcoming spring season.

## Supplementary Material

1
