# Supplementary material for: Optimizing COVID-19 control with asymptomatic surveillance testing in a university environment
Source: medRxiv. 2021 Oct 27:2020.11.12.20230870. Preprint. [Version 3] doi: 10.1101/2020.11.12.20230870 (PMC7805470; doi:10.1101/2020.11.12.20230870)
Supplement: 1 [file NIHPP2020.11.12.20230870V3-supplement-1.pdf]

Supplementary Appendix.

Text S1. ***Model Description.***

Our publicly-available Github repository (1) provides opensource code to reproduce all simulations and analyses presented in our paper. We summarize the practical implementation details of our modeling design for ease-of-access here.

Our model takes the form of a stochastic branching process model, in which a subset population of exposed individuals (0.5%, derived from the mean percentage of positive tests in our UC Berkeley community (2)) is introduced into a hypothetical 20,000 person community that approximates the campus utilization goals for our university in spring 2021. The model code builds up to a single function `replicate.epidemic()` which runs a specified number of stochastic simulations from a defined parameter set, using the function 'simulate.epidemic()'. Within the 'simulate.epidemic()' function, we first construct a population of 20,000 persons in the sub-function, 'initiate.pop()'. Within this initiation function, each person in our population is individually numbered, assigned a viral titer trajectory that will be followed if that individual becomes infected (Text B), and assigned a suite of disease metrics drawn stochastically from a specified set of parameter distributions, as outlined in Text S3.

Text S2. ***Within-host viral dynamics***

<u>*Titer Trajectories.*</u>

For computational efficiency, we pre-generated 20,000 50-day individual titer trajectories and saved them as an .Rdata file, `"titer.dat.20K.Rdata"`. To generate these trajectories, we used a within-host viral kinetics model structured after the classic target cell model (3–5). Code for this model is available in the 'model-sandbox' folder of our Github release, under file `viral-load.R`, which iterates the following simple model and parameter values derived from Ke et al. (2020), describing the dynamics of SARS-CoV-2 proliferation in the upper respiratory tract (6):

$$\frac{dT_C}{dt} = -\beta T_C V$$

$$\frac{dE}{dt} = \beta T_C V - kE$$

$$\frac{dI}{dt} = kE - \delta I$$

$$\frac{dV}{dt} = pI - cV$$

where T_C corresponds to the target cell population, β is the transmission rate of free virus to target cell invasion, k corresponds to the inverse of the duration of the virus eclipse phase, and δ corresponds to the inverse of the incubation period of an infected cell. p then gives the burst size of a virus-infected cell and c equals the inverse of the lifespan of free virus subject to natural

virus mortality and immune predation. Parameter values used to generate each titer trajectory (with a standard deviation of .1x the value of each parameter introduced to add stochasticity in each iteration) are derived from Ke et al. (2020) (6), after fitting this model to individual patient data tracking viral loads through time in the upper respiratory tract of SARS-CoV-2-infected individuals:

starting conditions: $T_C = 4 * 10^6; E = 0; I = 1; V = 0$
parameter values: $\beta = 1.9 * 10^{-6}; k = 4; c = 10; \delta = 1.9; p = 51.4$

Note that Ke et al. (2020) (6) also explore the within-host dynamics of SARS-CoV-2 infection in the lower respiratory tract; however, since we model human-to-human transmissibility as inferred by viral load in nasopharyngeal swab samples (which better reflect the viral load in the upper respiratory tract), we ignore the lower respiratory dynamics here.

<u>*Infectivity by Viral Load.*</u>
After Ke et al. (2020) (6), we next estimated the probability of infection given contact at a specific viral load, using a Michaelis-Menton-like function. Following Ke et al. (2020), we described the probability this probability as:

$$P(transmission) = 1 - \exp\left(-1 * \left(\theta\left(\frac{V}{V + K_m}\right)\right)\right)$$

where K_m corresponds to the saturation constant by which proportional gains in infectiousness with viral load diminish at increasingly high viral titers and θ is a constant, such that the maximum transmission capacity at any moment equals $1 - e^{-\theta}$. Ke et al. (2020) modeled a constant hazard of contact events for infectious individuals and therefore fixed θ at a value of 0.05, corresponding to a ~5% probability of a given contact resulting in transmission. Because we draw possible transmissions events from a negative binomial SARS-CoV-2 R_0 distribution, (mean= 2.5 and k=0.10 (7)) but ultimately know that R_E for our university environment should have a value of just above one (8), we instead fixed θ at a value of 0.72, corresponding to a ~51% probability of a given contact resulting in transmission, thus effectively halving R_0 to generate R_E. The exact probability varied as a function of the timing of each contact event across the trajectory of within-host viral load, with transmissions favored earlier in an infection trajectory when viral load peaks (9).

Text S3. *Individual disease metrics*
Figures in our paper are derived from 100x replications of each set of parameter values, which we manipulate to explore a range of non-pharmaceutical interventions (NPIs) to combat COVID-19 dynamics in our system. Our flexible model allows for the introduction of NPIs for COVID-19 control in four different forms: (1) group size limits, (2) symptom-based isolations, (3) surveillance testing isolations, and (4) contact tracing isolations that follow after cases are identified through screening from symptomatic or surveillance testing. These interventions

modify the suite of disease metrics drawn upon model initiation for each numbered individual in the dataset. We summarize the disease metrics drawn at initiation for all members of the population here:

- **Time of next test:** allocated based on the selected asymptomatic surveillance testing regime. We assume the week starts with day 1 on Saturday and day 7 on Friday. If n.test.days =2, then tests are distributed on Monday (day 3) and Friday (day 7) of each week. As timesteps advance and individuals reach their respective test days, the next test day is updated based on the testing regime (if semi-weekly, the next test day is advanced 3 days; if weekly, the next test day is advanced 7 days; if every-two-weeks, the next test day is advanced 14 days).
- **Beginning/end time of test sensitivity:** based on test limit of detection (LOD) as specified at model outset, this corresponds to the timestep post exposure at which an individual viral titer crosses the threshold for being detectable by the chosen test, both as titers increase at the beginning of a disease trajectory and decrease at the end.
- **Adherence with testing regime:** Y/N, allocated randomly across individuals based on the proportion of the population modeled as complying with the surveillance testing intervention (90% of individuals in all scenarios modeled in our paper).
- **Adherence with group limit**: Y/N, allocated randomly across individuals based on the proportion of the population modeled as complying with the group size limits imposed at outset (90% of individuals in all scenarios modeled in our paper; see 'number of potential onward cases generated for' for how group size interacts with cases).
- **Adherence with contact tracing regimen**: Y/N, allocated randomly across individuals based on the proportion of the population modeled as complying with the contact tracing intervention imposed at outset (90% of individuals in all scenarios modeled in our paper).
- **Time of symptom onset**: determined by randomly drawing a titer limit for symptom onset for each individual from a lognormal distribution with a mean of 1e+05 cp/μl RNA and a standard deviation of 1e+04 cp/μl (10–12). The timing of symptom onset then corresponds to the time post-exposure at which each individual's titer trajectory crosses the corresponding titer limit. According to this approach, under default parameter values, symptom onset occurred between 2 to 4 days post-exposure in our model, and ~32% of the population never presented with symptoms at all (Fig. 1, main text).
- **Time of symptom-based isolation:** based on delay lag post-symptom onset, drawn from a lognormal distribution with a mean of the specified number of days of symptom isolation lag (1-5 or infinity) and a standard deviation of 0.5 days.
- **Time of tracing-based isolation**: based on contact tracing lag for those adhering to the contact tracing regimen in place. Parameter must be updated with each timestep until individual becomes infected; value then becomes fixed at time of infector isolation, plus corresponding lag drawn from a lognormal distribution with a mean of one day and a standard deviation of 0.5 days.

- **Time of testing-based isolation:** based on turnaround time to isolation post testing, drawn from a lognormal distribution with a mean of the specified number of delay days (1-5, 10, or infinity) and a standard deviation of 0.5 days. Parameter is updated when 'time of next test' is updated for each individual in our model.
- **Disease status:** 'susceptible' = 0, 'exposed' = 3, 'infectious' = 1, 'recovered' = 5, 'vaccinated'= 6. At onset, all individuals are modeled as susceptible, excepting the 0.5% which are introduced as infectious (1) to seed the epidemic and the 'prop-vaccinated', a parameter encoding the proportion of the target population that is vaccinated prior to the start of epidemic simulations. We additionally encode a 'prop-breakthrough' parameter which corresponds to the proportion of vaccinated individuals who experience breakthrough infections. In simulations presented in our paper, 95% of vaccinated individuals are treated as if fully immune, while 5% of individuals experience breakthrough infections; these breakthrough cases are modeled stochastically, based on probability at the timestep in which each possible infection encounter occurs.

Number of potential onward cases generated: Several figures in the main text of our manuscript present the R_E reduction capacity of a specified intervention, which we calculate as the difference between the average of the number of potential onward cases generated and the number of actual onward cases generated for each individual after an intervention is adopted. To compute the number of potential onward cases generated for each individual, we first draw a number of possible cases from a negative binomial distribution with a mean of 2.5 and a dispersion parameter (k) of 0.10, as estimated for SARS-CoV-2 (7) (or with a mean of 6 in later simulations to represent the heightened transmissibility of the Delta variant (13)). Next, we assume that a minority of transmission events will be lost to the external environment through contacts between UC Berkeley students and members from the outside community. We do not track these 'lost cases' but instead simply reduce the total number of potential onward cases to the proportion constrained within UCB: 90% in simulations presented in the main text and 50% in the sensitivity analysis presented in Fig. S5.

Then, we draw a number of possible onward transmission events for the remaining cases for each infectious individual from a simple Poisson distribution with $\lambda = 3$, signifying the average number of possible encounters (i.e. cross-household dining, shared car rides, indoor meetings, etc.) per person that could result in transmission. We then distribute each infectious person's original number of R_0-derived potential cases among these events at random, ensuring that multiple transmissions are possible at a single event; the most extreme superspreading events thus occur when persons with heterogeneously high infectiousness draw a large number of potential cases, which are concentrated within a relatively small number of discrete transmission events. For example, if an infectious individual draws an R_0 value of 16 and an event number value of 4, then those 16 potential infections are randomly distributed among 4 events.

Next, we use published estimates of the generation time of onward transmission events for SARS-CoV-2 infection to draw event times for each event, based on a weibull distribution with a shape parameter = 2.826 and a scale parameter = 5.665, as specified in Ferretti et al. (2020) (9). Following the above example, 4 discrete generation times would be assigned to cases across the 4 pre-allocated events.

Since each individual is already pre-assigned a within-host viral titer trajectory in our modeling framework, we next examine the viral load specified at the generation time of each transmission event and determine if each case assigned to that event actually occurs. Each case is considered individually, and the probability of transmission is computed stochastically based on the value of the individual's viral titer at the time of the event (higher titer infections are more likely to generate onward transmission events) (Text S2). In the above example, all 16 possible transmissions would be individually assessed, though several would have the same titer, corresponding to the infectious person's titer at the time point of each contact event (4 possible). Since our maximum probability of a case occurring at max viral load is ~51% (Text S2), our original R_0-derived cases are here halved, resulting in an average of 1.05 onward transmission events per infectious individual (or just under 3 in the case of Delta simulations) in the absence of the NPIs examined here (but reflecting social distancing and mask wearing), which, as specified in the main text, is in line with current estimates from Alameda County, CA (8).

For the purposes of our example, let's assume that 10 of those possible 16 cases occur, allocated across 4 different events, with 7 cases at one event and one case each at 3 other events.

- **Number of actual onward cases generated**: From the number of possible cases generated, we next apply the relevant intervention and iterate forward in time to determine the actual number of cases generated by each infectious individual across the time course of our modeled epidemics. For symptom and surveillance testing-based isolations, as well as contact tracing, no cases are generated if an infectious individual is isolated prior to the generation time of any possible onward cases. For NPIs in the form of group size limits, case reduction in our model is performed prior to the initiation of the epidemic time series, and case numbers for each transmission event are truncated at the intervention limit.

Again following the example listed above, if we imagine that the imposed group size limit is 6, then the 7 cases assigned to a single event will be truncated to 6, meaning that 9 out of the 10 potential cases are allowed to occur after the intervention. Our model is conservative in assessing the impact of a group-size intervention by allowing some portion of those superspreading cases to occur, rather than assuming that a group size limit-abiding infectious individual does not attend larger-than-allowable events altogether. Because only 90% of the population adheres to group size intervention in any given simulation (or 50% in sensitivity analyses; see Fig. S1), some proportion of large superspreading events will still take place at random, even after NPIs are imposed.

Following onset of infection, the timings of symptom-, tracing-, and asymptomatic testing-based isolations are then compared and the earliest time is selected as the actual mechanism (if any) of isolation for that individual. The number of actual onward cases generated is then updated if isolation occurs prior to some new case generations. Additionally, all individuals identified as infectious are additionally assigned the following metrics:

- **Isolation time of infector**
- **Source of infection** (external Alameda County vs. UC Berkeley community member)
- **ID number of infector,** if from UC Berkeley

The cycle then repeats in the next timestep when all "actual infections" for each infectious individual are then assigned to new susceptible individuals. The epidemic continues with updated parameters for all newly exposed individuals until either the end of the time series is reached or no more susceptible individuals remain in the population.

References for Supplementary File 1.

1. Brook CE, Northrup GR, Boots M (2020) Code for "Optimizing COVID-19 control with asymptomatic surveillance testing in a university environment." doi:10.5281/zenodo.4131223.
2. UC Berkeley COVID-19 Dashboard Available at: https://coronavirus.berkeley.edu/dashboard/?utm_source=Response+and+Recovery&utm_campaign=5247da06c4-Response_Recovery_2020_10_09&utm_medium=email&utm_term=0_940930e328-5247da06c4-389116456 [Accessed October 1, 2020].
3. Perelson AS (2002) Modelling viral and immune system dynamics. *Nat Rev Immunol* 2(1):28–36.
4. Ho DD, et al. (1995) Rapid turnover of plasma virions and CD4 lymphocytes in HIV-1 infection. *Nature* 373:123–126.
5. Nowak MA, May RM (2000) *Virus Dynamics: Mathematical Principles of Immunology and Virology* (Oxford University Press, Oxford, UK).
6. Ke R, Zitzmann C, Ribeiro RM, Perelson AS (2020) Kinetics of SARS-CoV-2 infection in the human upper and lower respiratory tracts and their relationship with infectiousness. *medRxiv*:2020.09.25.20201772.
7. Endo A, Abbott S, Kucharski AJ, Funk S (2020) Estimating the overdispersion in COVID-19 transmission using outbreak sizes outside China. *Wellcome Open Res* 5:67.
8. Schwab J, Balzer LB, Geng E, Peng J, Petersen ML Local Epidemic Modeling for Management and Action. Available at: https://localepi.github.io/LEMMA/.
9. Ferretti L, et al. (2020) Quantifying SARS-CoV-2 transmission suggests epidemic control with digital contact tracing. *Science* 368(6491):eabb6936.
10. Wölfel R, et al. (2020) Virological assessment of hospitalized patients with COVID-2019. *Nature* 581(7809):465–469.
11. Quicke K, et al. (2020) Longitudinal surveillance for SARS-CoV-2 RNA among asymptomatic staff in five Colorado skilled nursing facilities: Epidemiologic, virologic and sequence analysis. *medRxiv*. doi:10.1101/2020.06.08.20125989v1.
12. La Scola B, et al. (2020) Viral RNA load as determined by cell culture as a management tool for discharge of SARS-CoV-2 patients from infectious disease wards. *Eur J Clin Microbiol Infect Dis* 39(6):1059–1061.
13. Liu Y, Rocklov J (2021) The reproductive number of the Delta variant of SARS-CoV-2 is far higher compared to the ancestral SARS-CoV-2 virus. *J Travel Med* 3(27):584–586.

Supplementary Figures

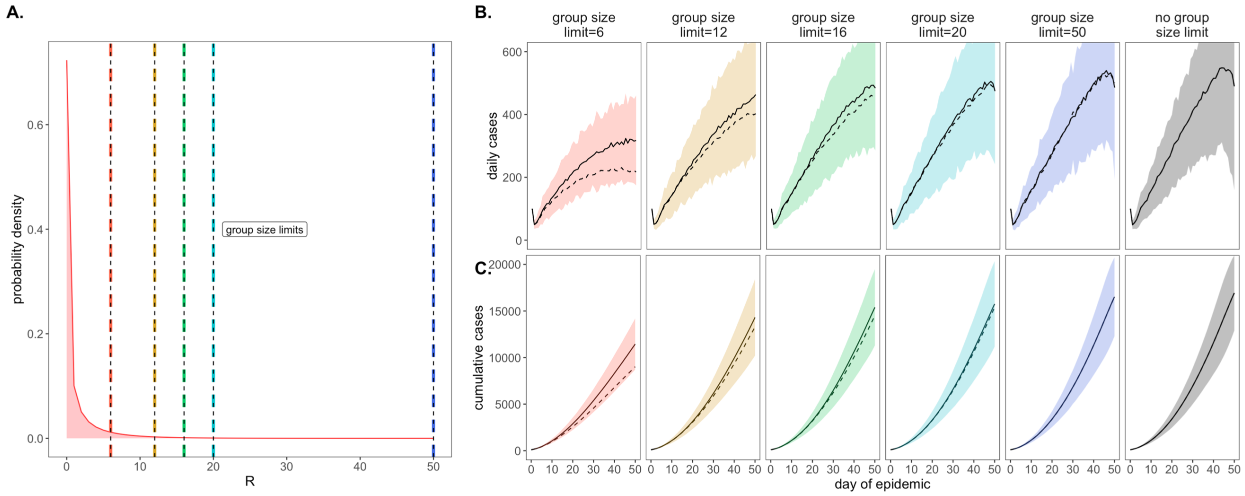

Figure S1. Figure replicates Fig. 2 (main text) assuming only 50% adherence to group size limitations vs. the 90% adherence presented in the main text. **A.** Negative binomial R_E distribution with mean = 1.05 and dispersion parameter (k) = 0.10. The colored vertical dashes indicate group size limits that 'chop the tail' on the R_E distribution; for 90% of the population, coincident cases allocated to the same transmission event were truncated at the corresponding threshold for each intervention. **B.** Daily new cases and, **C.** Cumulative cases, across a 50-day time series with 95% confidence intervals by standard error depicted under corresponding, color-coded group size limits. Mean output of simulations under 50% adherence are shown as solid black lines, with the dashed line corresponding to mean output under the 90% adherence assumptions presented in the main text.

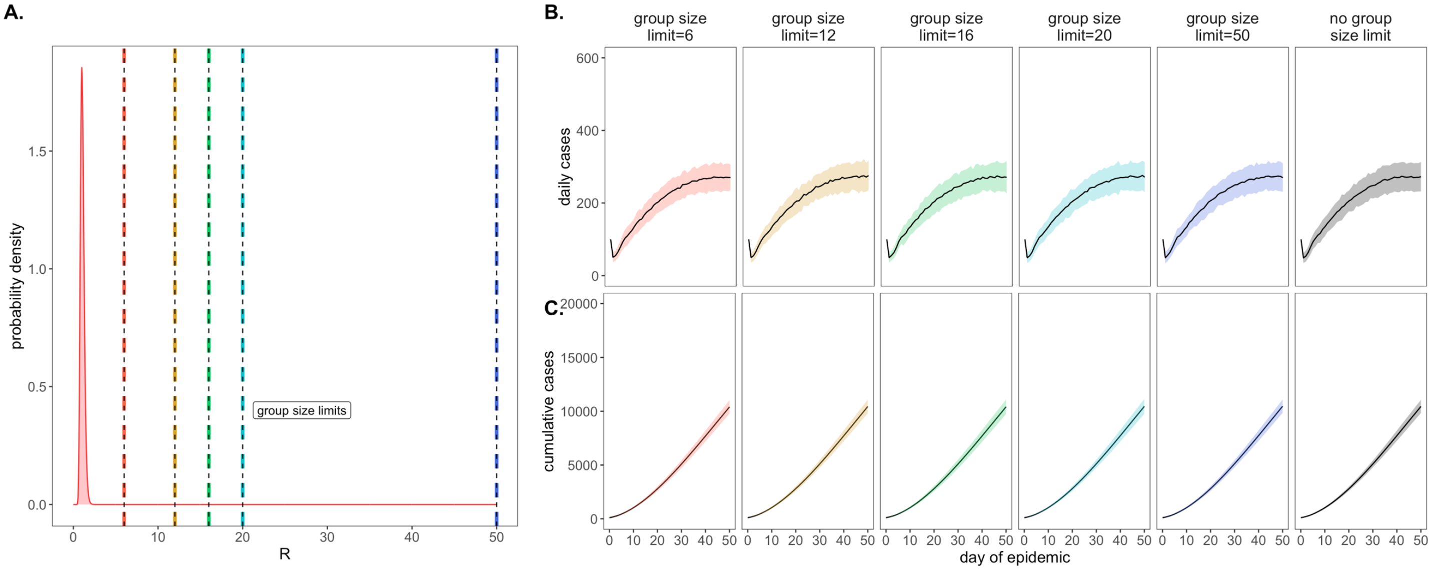

Figure S2. Figure replicates Fig. 2 (main text) at a log-normal distribution for R_E, instead of negative binomial. **A.** Log-normal R_E distribution with a mean of 1.05 and a standard deviation of 1.233. The colored vertical dashes indicate the group size limits that 'chop the tail' on the R_E distribution. **B.** Daily new cases and, **C.** cumulative cases, across a 50-day time series under corresponding, color-coded group size limits.

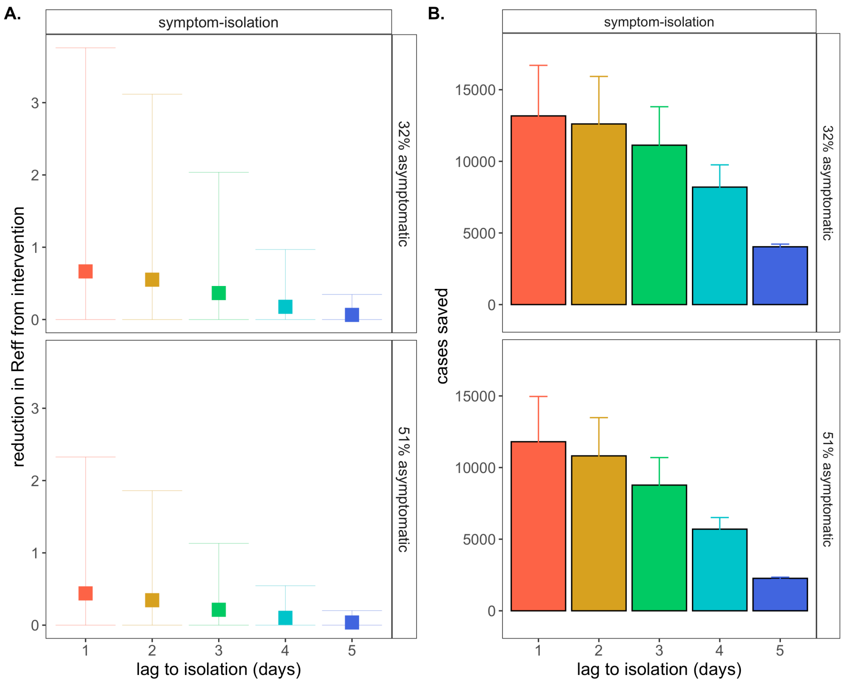

Figure S3. Figure replicates symptom-isolation panels from Fig. 3 (main text) in top row, showing **A.** mean reduction in R_E and **B.** cumulative cases saved across 50-day simulated epidemics under differing lag times to isolation, assuming a threshold titer for symptom onset by which ~32% of the population presents as asymptomatic. A comparison at a titer threshold for which ~51% of the population presents as asymptomatic demonstrates how a higher proportion of asymptomatic individuals in the population erodes the effectiveness of the symptom-based isolation intervention; asymptomatic status has no impact on the effectiveness of group size limits or asymptomatic surveillance testing interventions.

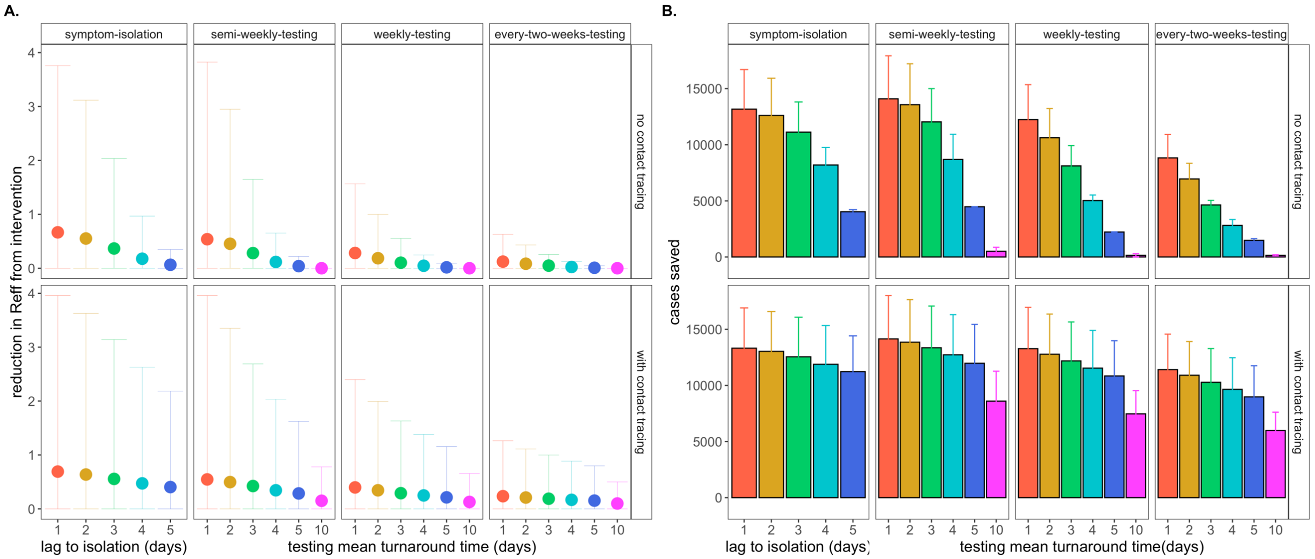

Figure S4. Figure replicates symptom-isolation panels from Fig. 3 (main text) in top row, showing **A.** mean reduction in R_E and **B.** cumulative cases saved across 50-day simulated epidemics for NPIs of both symptom-based and testing-based isolation, across a range of different lag times or turnaround times to isolation (for, respectively symptom- or testing-based isolations). All testing-based interventions depicted are shown at a limit of detection=10^1 cp/µl. In the bottom row, **A.** mean reduction in R_E and **B.** cumulative cases saved are depicted for a comparative intervention which adds an additional single-day lag in contact tracing to the respective symptom-based or testing-based isolation. Under these combined interventions, even previously ineffective testing interventions with 10-day turnaround time show gains beyond no intervention at all.

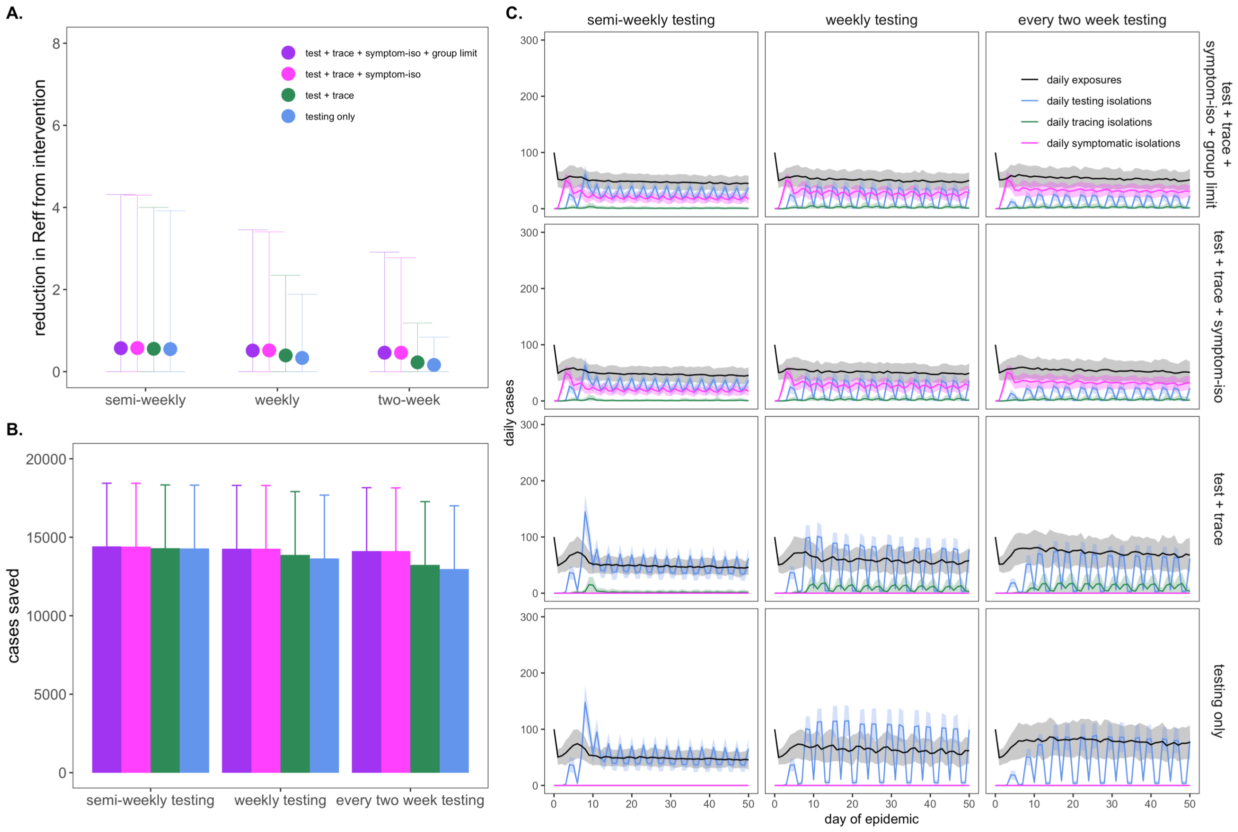

Figure S5. Figure replicates Fig. 4 of the main text, under assumptions of 50% of cases lost to the outside community, as compared to the 10% modeled in the main article. **A.** Mean reduction in R_E, **B.** cumulative cases saved, and **C.** daily case counts for the first 50 days of the epidemic, across regimes of differing testing frequency and a combination of surveillance testing, contact tracing, symptomatic isolation, and group size limit interventions. All scenarios depicted here assumed test turnaround time, symptomatic isolation lags, and contact tracing lags drawn from a log-normal distribution with mean=one day. Limit of detection was fixed at 10^1 and group size limits at 12. Dynamics shown here are from simulations in which testing was limited to two test days per week. NPIs have proportionally less impact on R_E reduction (A) but nonetheless manage to avert an equal number of cases (B) when the university is modeled as a more open, community-integrated environment. Under this scenario, interventions function primarily to isolate cases from the external environment, rather than curb onward, within-community transmission. For this reason, daily variance in exposure rate is also diminished under assumptions of a higher proportion of transmissions lost to the surrounding community.
**Note: R_E reduction (panel A) is calculated as the difference in mean R_E in the absence vs. presence of a given NPI. The upper confidence limit (uci) in R_E reduction is calculated as the difference in uci R_E in the absence vs. presence of NPI. In our model, mean R_E in the absence of NPI equals 1.05 and uci R_E in the absence of NPI equals 8.6.*

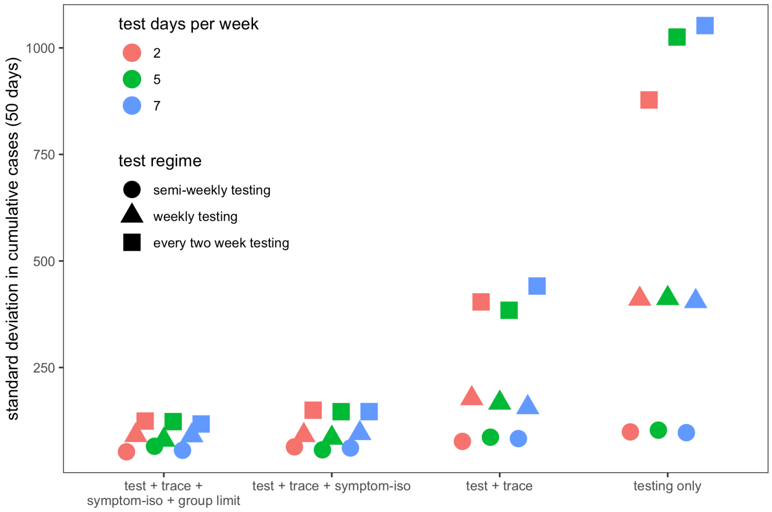

Figure S6. Figure extends results from Fig. 4 (main text), showing the standard deviation in cumulative cases from 50-day simulated epidemics, across regimes of differing testing frequency and a combination of surveillance testing, contact tracing, symptomatic isolation, and group size limit interventions. All scenarios depicted here assume test turnaround time, symptomatic isolation lags, and contact tracing lags drawn from a log-normal distribution with mean=1 day. Limit of detection is fixed at 10^1 and group size limits at 12. Dynamics compare tests of differing frequency (semi-weekly, weekly, every two weeks) distributed across variable numbers of days in a given week (2,5,7). Additional layers of intervention and more testing days per week reduce the standard deviation in cumulative cases.

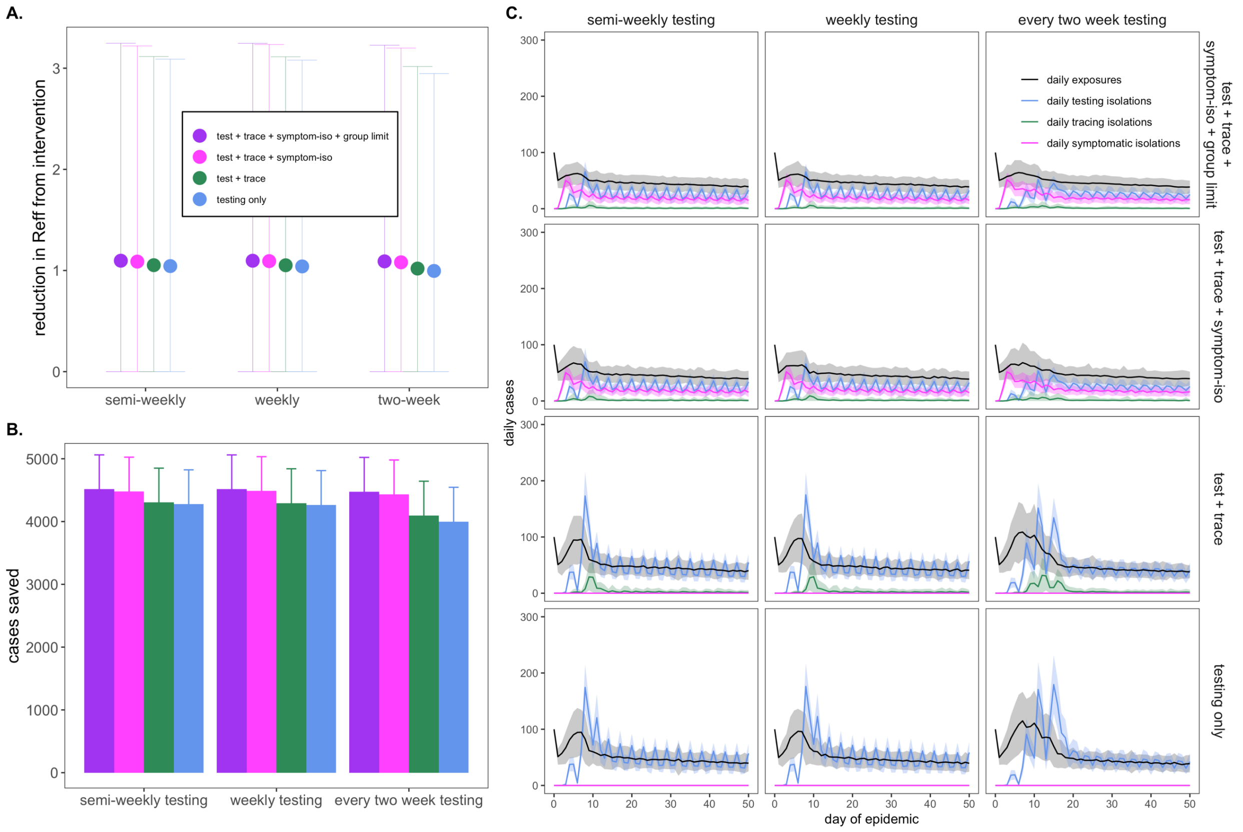

Figure S7. Figure largely replicates Fig. 4 of the main text, under assumptions of mean R_0 = 6 and 60% of the baseline campus population vaccinated, approximating circulation of the Delta variant in the undergraduate population of the University of Alabama, Tuscaloosa at the time of this writing. Note that y-axes for panel **A.** and **B.** differ from those depicted in Fig 4 of the main text and from Fig. S8 below. **A.** Mean reduction in R_E and **B.** cumulative cases saved compared to a baseline scenario in which no behavior-based or testing NPIs were applied but simulations were run under assumptions of 60% vaccination in an R_0=6 environment. **C.** Daily case counts for the first 50 days of the epidemic, across regimes of differing testing frequency and a combination of surveillance testing, contact tracing, symptomatic isolation, and group size limit interventions. All scenarios depicted here assumed test turnaround time, symptomatic isolation lags, and contact tracing lags drawn from a log-normal distribution with mean=one day. Limit of detection was fixed at 10^1 and group size limits at 12. Dynamics shown here are from simulations in which testing was limited to two test days per week. Combined, asymptomatic surveillance testing and behavior-based NPIs still reduce R_E and avert cases but impacts are reduced compared to no vaccination settings (main text) because fewer opportunities for infection arise. Variance between simulations and interventions is also diminished in this more mild epidemic scenario, indicating that testing alone, without rigorous extensive additional interventions, can effectively control outbreaks.

**Note: R_E reduction (panel A) is calculated as the difference in mean R_E in the absence vs. presence of a given NPI. The upper confidence limit (uci) in R_E reduction is calculated as the difference in uci R_E in the absence vs. presence of NPI. In our model, mean R_E under Delta variant transmission assumptions in the absence of NPIs, but including 60% population-level vaccination, equals 1.12 and uci R_E equals 3.33.*

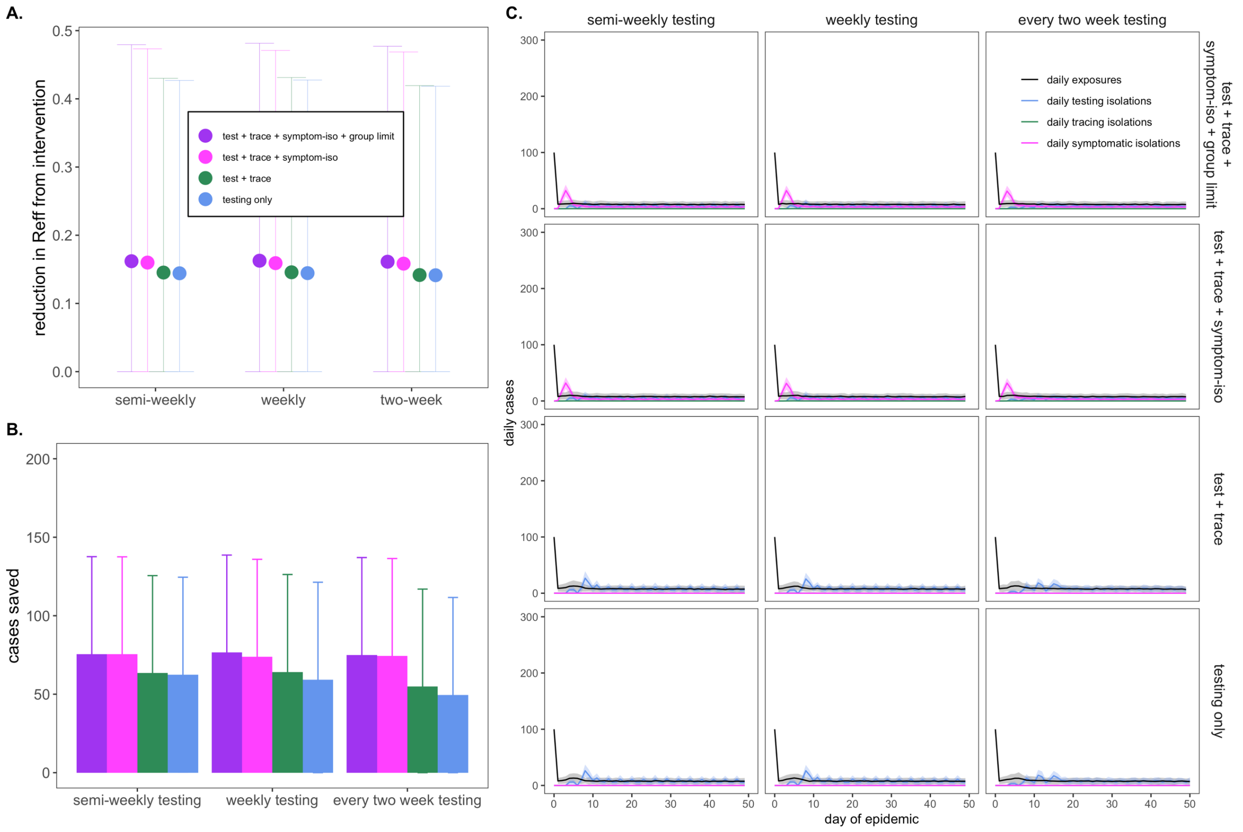

Figure S8. Figure largely replicates Fig. 4 of the main text, under assumptions of mean R_0 = 6 and 97.7% of the baseline campus population vaccinated, approximating circulation of the Delta variant in the undergraduate population of UC Berkeley at the time of this writing. Note that y-axes for panel **A.** and **B.** differ from those depicted in Fig 4 of the main text and from Fig. S7 above. **A.** Mean reduction in R_E and **B.** cumulative cases saved compared to a baseline scenario in which no behavior-based or testing NPIs were applied but simulations were run under assumptions of 97.7% vaccination in an R_0=6 environment. **C.** Daily case counts for the first 50 days of the epidemic, across regimes of differing testing frequency and a combination of surveillance testing, contact tracing, symptomatic isolation, and group size limit interventions. All scenarios depicted here assumed test turnaround time, symptomatic isolation lags, and contact tracing lags drawn from a log-normal distribution with mean=one day. Limit of detection was fixed at 10^1 and group size limits at 12. Dynamics shown here are from simulations in which testing was limited to two test days per week. Even in highly vaccinated university settings, behavior-based NPIs and asymptomatic surveillance testing reduce R_E and avert cases largely derived from breakthrough infections, though lower baseline case counts equate to lower gains in R_E reduction and case aversions. Variance between simulations and between interventions is most diminished in this epidemic scenario, indicating that testing alone, without rigorous extensive additional interventions, can effectively control outbreaks.

**Note: R_E reduction (panel A) is calculated as the difference in mean R_E in the absence vs. presence of a given NPI. The upper confidence limit (uci) in R_E reduction is calculated as the difference in uci R_E in the absence vs. presence of NPI. In our model, mean R_E under Delta variant transmission assumptions in the absence of NPIs, but including 97.7% population-level vaccination, equals 0.17 and uci R_E equals 0.51.*

Legends for Datasets S1-S3.

Dataset S1. Averaged total cases saved and mean R_E reduction across group size limit, symptomatic isolation, and surveillance testing NPIs. Summarized model output from 100x simulations across all NPIs presented in Fig. 2 and Fig. 3, main text. Confidence intervals represent 1.96*standard deviation in case reduction or R_E reduction.

Dataset S2. Averaged total cases saved and mean R_E reduction across symptomatic isolation, and surveillance testing NPIs, under regimes with and without contact tracing. Summarized model output from 100x simulations across all NPIs presented in SI-Appendix, Fig. S3.

Dataset S3. Averaged total cases saved and mean R_E reduction across combined intervention approaches. Summarized model output from 100x simulations across all NPIs presented in Fig. 4, main text.

All other model output available as saved .Rdata files in our publicly-available Github repository:

Brook CE, Northrup GR, Boots M (2020) Code for "Optimizing COVID-19 control with asymptomatic surveillance testing in a university environment." doi:10.5281/zenodo.4131223
